# Strong Relationships in Acid-Base Chemistry – Modeling Protons Based on Predictable Concentrations of Strong Ions, Total Weak Acid Concentrations, and pCO_2_

**DOI:** 10.1371/journal.pone.0162872

**Published:** 2016-09-15

**Authors:** Troels Ring, John A. Kellum

**Affiliations:** 1 Department of Nephrology. Aalborg University Hospital. Aalborg 9000, Denmark; 2 The Center for Critical Care Nephrology. Department of Critical Care Medicine, University of Pittsburgh School of Medicine, and University of Pittsburgh Medical Center, Pittsburgh, PA, United States of America; Kaohsiung Medical University, TAIWAN

## Abstract

Understanding acid-base regulation is often reduced to pigeonholing clinical states into categories of disorders based on arterial blood sampling. An earlier ambition to quantitatively explain disorders by measuring production and elimination of acid has not become standard clinical practice. Seeking back to classical physical chemistry we propose that in any compartment, the requirement of electroneutrality leads to a strong relationship between charged moieties. This relationship is derived in the form of a general equation stating charge balance, making it possible to calculate [H^+^] and pH based on all other charged moieties. Therefore, to validate this construct we investigated a large number of blood samples from intensive care patients, where both data and pathology is plentiful, by comparing the measured pH to the modeled pH. We were able to predict both the mean pattern and the individual fluctuation in pH based on all other measured charges with a correlation of approximately 90% in individual patient series. However, there was a shift in pH so that fitted pH in general is overestimated (95% confidence interval -0.072–0.210) and we examine some explanations for this shift. Having confirmed the relationship between charged species we then examine some of the classical and recent literature concerning the importance of charge balance. We conclude that focusing on the charges which are predictable such as strong ions and total concentrations of weak acids leads to new insights with important implications for medicine and physiology. Importantly this construct should pave the way for quantitative acid-base models looking into the underlying mechanisms of disorders rather than just classifying them.

## Introduction

A natural starting point in understanding acid-base is to seek an explanation that pH in any specified fluid has exactly the observed value. If that is possible, mechanisms in acid-base disorders can be formulated in terms of the conditions determining pH. Looking back, this ambition is not new.

Brown et al [1] wrote in 1989 “In analysis of acid-base balance, one must keep in mind the fact that electroneutrality dictates that the sum of the charges of the nonreactive ions in urine ([Na^+^]+[K^+^]-[Cl^-^]) must be equal and opposite in signs to the sum of charges of buffer ions plus organic ions. Thus the contribution of urinary excretion to systemic acid-base balance can be assessed either by measuring the buffer plus organic ions or the nonreactive ions in urine”.

A similar insight was demonstrated by Lemann et al [2] reporting a strong effect whereby a negative whole body balance of [Na^+^]+[K^+^]-[Cl^-^] yielded a strongly positive acidifying effect (Figure 3 in reference [2]).

Already in 1914, Spiro and Koppel [3] noticed that in contrast to electrolytes and total concentrations of weak acids and bases, protons and hydroxyl ions are not additive in fluids with a pH observed in plasma or urine, and wrote equations, based on the principle of charge balance, from which to deduce the [H^+^]. Although these insights were confirmed [4,5]the traditional discourse in acid-base has taken another path. Hence, noting that hydrogen is ubiquitously produced in metabolism [6] quantitative accounting of acid-base chemistry has been attempted indirectly in terms of summation of metabolites known to be involved in producing or neutralizing protons [7]. Although this is the textbook version of acid-base [8–10] it has never penetrated far into clinical practice. This may be because it is technically demanding in terms of the measurements needed.

Hence, there should be a call for understanding acid-base in terms of measurements, which are more practical. This paper proposes such an understanding in the form a statement of charge balance, examines the validity of this expression on a large clinical sample and, independently, with data from a seminal paper by Pitts and Alexander [11]. Finally some consequences of this paradigm for modeling acid-base are presented.

## Materials and Methods

### Models and equations

The derivation of the theoretical model is presented in complete form as supplementary material (S1 Text), but here, in an overview to facilitate understanding is first shown results to demonstrate in the simplest possible system the meaning of the statement that protons are not additive. Then the effect of a single monovalent weak acid as derived in [3–5,12,13] is presented.

Water dissociation

$$K´w=\left[H^{+}\right]*\left[OH^{-}\right]$$ (1)

Charge balance with SID

$$\begin{array}{l} SID+\;[H^{+}]-\;\frac{K\prime w}{\left[H^{+}\right]}=0\\ {}[H^{+}]=-\frac{SID}{2}+\sqrt{\left[{\left(\frac{SID}{2}\right)}^{2}+K\prime w\right]}=0 \end{array}$$ (2)

An initial theoretical experiment was done to demonstrate the meaning of the central statement that protons are not additive [3]and thereby motivate the entire framework for this study. The example employed Eq 2 with 2 fluids randomly sampled with SID between -3 mM and 3 mM which were mixed 1:1. The mean values of [H^+^] and pH were compared to the corresponding values in the mixture.

### Monovalent weak acid

$$\left[H^{+}\right]*\left[A^{-}\right]=k_{a}*\left[HA\right]\text{ dissociation of weak acid}$$ (3)

$$\left[Atot\right]=\left[HA\right]+\left[A^{-}\right]\text{ total concentration}$$ (4)

$$\left[A^{-}\right]=\frac{k_{a}*\left[Atot\right]}{k_{a}+\left[H^{+}\right]}\text{ calculated charge}$$ (5)

### Charge balance

$$\begin{array}{l} \left[H^{+}\right]+SID-\frac{K´w}{\left[H^{+}\right]}-\frac{K_{a}*Atot}{\left[H^{+}\right]+K_{a}}=0 \end{array}$$ (6)

Here SID (“strong ion difference”) is the difference between concentrations of totally dissociated base and totally dissociated acid, K´w is the water dissociation constant, K_a_ is the weak acid dissociation constant, and Atot is the total concentration of weak acid. SID constituents and total concentration of weak acids, Atot, can be directly measured and predicted, and from that, proton concentrations can be operationalized. Proton concentrations thereby are explicit functions of the other charged moieties, and indeed any missing entry can be found given the others.

Generalization of this relationship to include multiple buffers and also the effect of CO_2_ using the principles above is straightforward physical chemistry, yielding equations like Eq 7(for derivation and extension to multivariate buffers, see supplementary material S1 Text)

$$\begin{array}{l} \left[H^{+}\right]+SID-\frac{K´w}{\left[H^{+}\right]}-\frac{k_{c}*pCO_{2}}{\left[H^{+}\right]}-\frac{2*k_{3}*k_{c}*pCO_{2}}{{\left[H^{+}\right]}^{2}}-P*\left(1+\frac{k_{2}}{k_{2}+\left[H^{+}\right]}\right)-\\ Alb*AF+\left[H^{+}\right]*Alb*\left(\frac{AH}{k_{h}+\left[H^{+}\right]}\right)-\sum \frac{k_{a}*Atot}{k_{a}+\left[H^{+}\right]}+\sum \frac{\left[H^{+}\right]*k_{b}*Btot}{\left[H^{+}\right]*k_{b}+k_{w}}=0 \end{array}$$ (7)

The entries in Eq 7 are explained in Table 1.

**Table 1:** Explanation for symbols and constants.

Entry	Explanation	Value	Ref
SID	Strong ion difference		
k_w_	Water auto-ionization constant 2.39*10^-14^ mol^2^/l^2^	2.39*10^-14^ mol^2^/l^2^	[13]
k_c_	Combined equilibrium and solubility constant for CO2	2.45*10^-11^ mol^2^/(l^2^*mmHg)	[13]
K_3_	Second dissociation constant of carbonic acid	5.76*10^-11^ mol/l	[13]
Alb	Molar albumin concentration		
AF	Number of fixed neg charges pr molecule of albumin	21	[13]
AH	Number of histidine residues pr molecule of albumin	16	[13]
k_h_	Histidine dissociation constant	1.77*10^-7^ mol/l	[13]
P	Total phosphate concentration		
k_2_	Second dissociation constant for phosphoric acid	6.8	
Atot, Btot	Total concentration of weak acid and base		
k_a_, k_b_	Dissociation constant for weak acid and base		

As indicated, the effect of phosphate, P, is focused on the second dissociation since in most biological fluids, including the first and third dissociation constant is inconsequential (but this issue is treated in separate analyzes provided in S1 Text).

While Eq 7 is derived from basic physical chemistry and is mathematically sound it does not necessarily have much practical utility either for physiology or medicine. Hence, SID is calculated based on a number of measurements, each with imprecision and likewise all other concentrations and dissociation constants are known only approximately. Above that, the physical chemistry assumes behaviour of say Na^+^ activity relative to measured [Na] which is difficult to verify [14]. Therefore, besides modelling directly in terms of measured concentrations, we also employ Davies’ modification [15] of the Debye-Hückel theory to calculate activity coefficients.

To find activity coefficients, ionic strength was obtained from $I=0.5*\sum c*Z^{2}$ and then $\text{log}(\gamma_{i})=-A*Z_{i}^{2}*\left(\frac{\sqrt{I}}{1+\sqrt{I}}-0.2*I\right)$, with c the vector of molar concentrations, and Z the charges. For the calculation of ionic strength, bicarbonate was calculated from the measured pCO_2_ and pH.

### Data sources and variable extraction

For empirical validation of Eq 7 we used data from critically ill patients with rapidly changing fluid, electrolyte and acid-base parameters. Importantly we chose to examine data from these patients for two reasons. First, they have frequent changes in fluid, electrolyte and acid-base status such that fluctuations in pH can be observed. It would unethical to induce such changes experimentally in humans. Second, because the unstable nature of this patient population, frequent measurements of arterial pH and the variables necessary to employ Eq 7 are made for clinical reasons.

We analyzed data on 41,852 adult patients admitted to any of 8 intensive care unit (ICUs) at the University of Pittsburgh Medical Center during an 8-year period (July 2000-October 2008). After excluding patients who received renal replacement therapy we searched the records electronically for patients that had at least 20 complete sets of data on SID constituents, phosphate, albumin, pCO_2_ and pH within their ICU stay. The study protocol was approved by the University of Pittsburgh Institutional Review Board. We used anonymized laboratory values obtained solely for clinical reasons. Therefore informed consent was not obtained.

Based on the times of measurements of arterial pCO_2_ and pH, we used linear interpolation of the other moieties over time so to have the best possible combined set of simultaneous values on which to model pH. Apart from lactate, pCO_2_ and pH, all measurements were made in venous plasma, but previous experience indicates that there may be minimal shift in the other variables [16]. Calcium and magnesium were both assumed to be 50% ionized.

### Validation procedures

Based on the 9 variables ([Na^+^], [K^+^], [Cl^-^], [Ca^++^], [Mg^++^], [Albumin], [P], [Lactate^-^], pCO_2_) pH was found applying Eq 7. The fitted pH was then compared with the measured pH in models with each patient as a random factor. The same procedure was employed including activity coefficients as described by Davies [15].

We then investigated possible sources of variance between measured and modeled pH using Eq 7 to find the concentration of an unknown buffer with an arbitrary pKa of 7, or if the fitted weak acid had a negative concentration, it was set to 0, and a weak base was fitted using a pKb of 6 to give a perfect fit. Importantly, Eq 7 does not distinguish between dependent and independent variables but simply, based on the concentrations of species involved, reconciles the fact that the charges sum to zero. Therefore, to find the unknown concentrations of weak acids that give a perfect fit, [H^+^] was calculated from the measured pH, and inserted in Eq 7 together with k_a_ and the other variables, leaving only one unknown: Atot, which was thereby found to solve the charge balance. Likewise, we examined how much SID had to change to yield perfect fit between modeled and measured pH.

Since the initial aim was to verify the ability of Eq 7 to predict [H^+^] in the range 10^-7^–10^-8^ based on moieties with measurement errors several orders larger, a theoretical sensitivity analysis was also employed to investigate if this could be possible at all. Since we had no replicate measurements obtained under controlled or identical circumstances, further sensitivity analysis was unrealistic.

### Comparisons and statistical procedures

To compare the different instances of modeled pH to the measured pH, we made scatterplots including the line of identity: x = y. Also we drew the line representing the population estimate as obtained from the hierarchical modeling employed. The latter is problematic [17] if the independent variable, measured pH, is not obtained without error. It was not considered feasible to perform Deming regression to compensate for the error in measured pH since this requires unavailable knowledge of the error ratio between modeled and measured variables [18]. Furthermore it was difficult to perform Deming regression respecting the hierarchical nature of the data. Therefore a Bland-Altman plot was made to illustrate the function of the concentration based modeling of pH in predicting measured pH, respecting the fact that the measurements were clustered, requiring a mixed model approach [19]. Furthermore, as a simplified illustration, simulation was performed under reasonable values of standard deviation of measured pH by the SIMEX procedure [20] directly on measured and estimated pH values [21], i.e. ignoring the hierarchical nature of data.

Finally we obtained prediction from a nonparametric kernel-based estimation [22] in which the clustering of measurements within patients was taken into account. Here the nonparametric model obtained from our measured and modeled values was used to predict fitted values based on measured values.

### Independent validation using published data

As an independent examination of the validity of the approach to understand pH, we examined data from the seminal paper by Pitts and Alexander [11]. 24 experiments were made on 6 dogs, reported in table I and II of their paper. Titratable acidity was measured as the amount of strong base added urine to obtain plasma pH, and reported together with flow, pH, and concentrations of phosphate and creatinine in final urine, and also plasma pH. From Eq 7 (ignoring moieties not modeled, and including creatinine with a K_b_ of 10^14-4.97^ (pKa 4.97 as assumed in Pitts and Alexander [11])) given urine [P], urine [creatinine] and pH we can find SID in urine, and with the same concentrations calculate the SID at plasma pH. Subtracting these and multiplying by flow gives an estimate of titratable acidity.

### Modeling procedures

All modeling was performed using R(3.2.1) [23] by simple root finding to Eq 7 with precision at least 10^-22^. The algorithm is robust and was verified to produce a single estimate in every case.

To take into account the dependency between data originating from each individual patient, a mixed effects modeling approach was used to compare measured with fitted pH. This procedure can be thought of as a generalization of linear regression where intercepts and perhaps slopes are allowed to vary by patient as random variables around a common population model [24,25].

Selected patient observations are summarized by mean and standard-deviation (SD).

## Results

### Initial theoretical experiment

**Fig 1:**
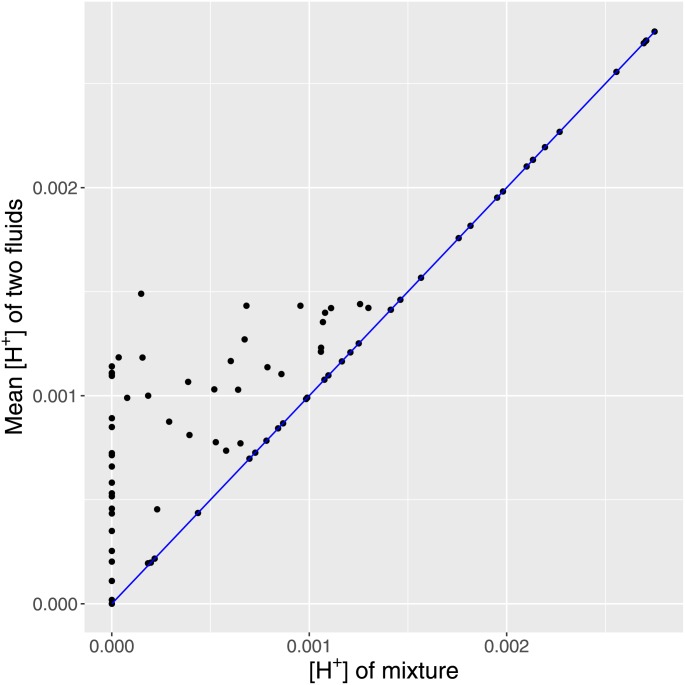
Fig 1 shows that [H^+^] in a fluid mixed 1:1 is not a simple function of the [H^+^] in the individual fluids. Blue line represents identity. The resulting [H^+^] is never higher than the mean [H^+^].

**Fig 2:**
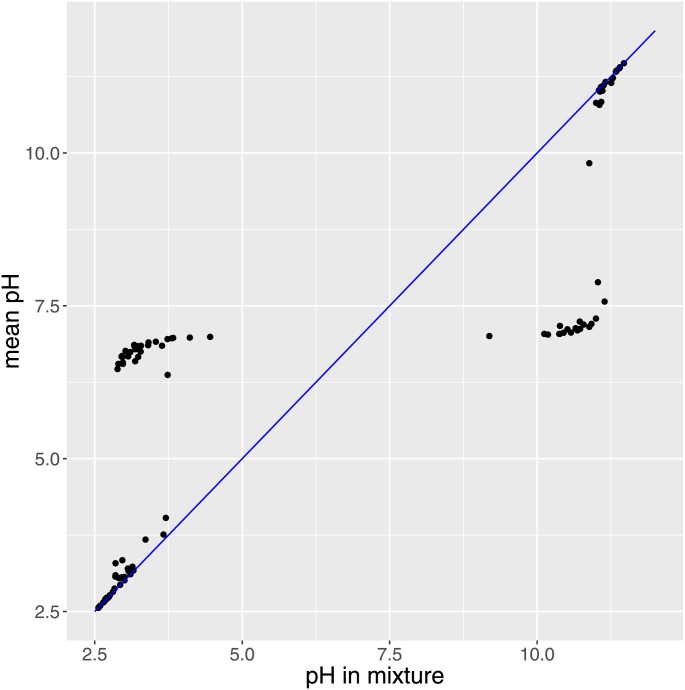
Fig 2 shows that pH in a 1:1 mixture is not predictable from pH in the constituent fluids.

Figs 1 and 2 demonstrate that [H^+^] in a mixture is never higher than the mean of [H^+^] in the two fluids, while the pH in a mixture is complicated related to the mean pH of the fluids. This forms the motivating fundament for understanding [H^+^] and pH in terms of all the charges present in the fluid.

### General data presentation

Only a small number of patients had complete sets of data with at least 20 time points. From these a few duplications also had to be removed. However, we successfully identified 2437 observations from 76 patients. Patients were selected only by the presence of data and not based on the values, and only the relationship between measured and modeled pH was of interest and therefore no patient information was extracted. Mean observed pH was 7.377 with SD 0.097 whereas mean modeled pH was 7.446 with SD 0.118. Mean pCO_2_ was 40.877 mmHg with SD 9.994 mmHg, mean SID was 36.884 mM with SD 5.621 mM, and mean [P] was 1.418 mM with SD 0.661 mM.

**Fig 3:**
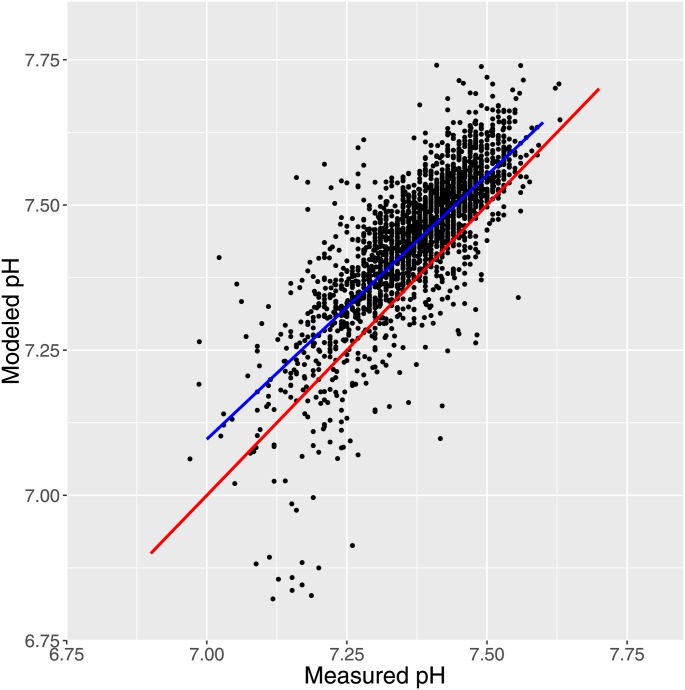
Fig 3 shows the overall fit for all data using a mixed model with random intercept. The thick blue line shows the population regression, the red line shows X = Y.

Taking all measurements together, fitted pH was found to be 0.732 + 0.909 * measured pH; with standard error of 0.099 and 0.013 on intercept and slope, respectively, with a mixed model fitting a random intercept; P<0.001 for both intercept and slope, Fig 3. Acknowledging the hierarchical structure yielded a better model (P<0.001), but fitting also a random slope gave no further improvement. Examination of residuals indicated no problems in the model.

**Fig 4:**
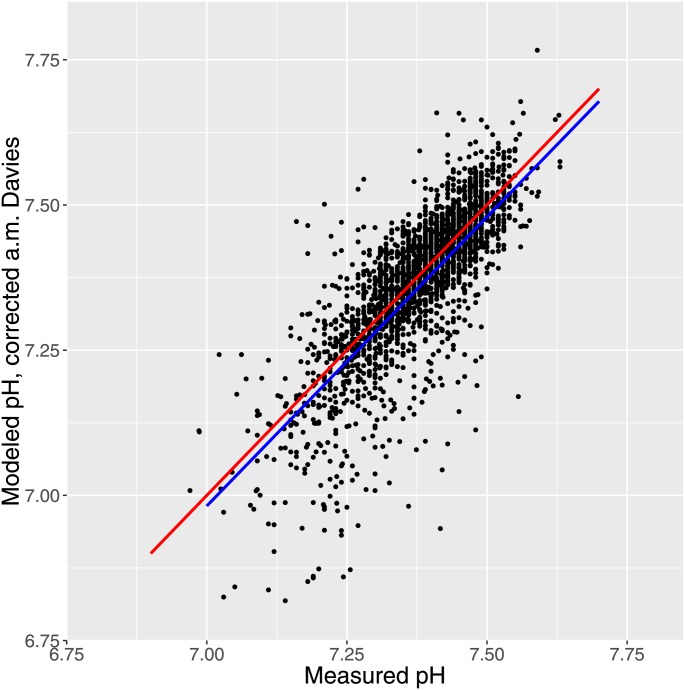
Fig 4 shows the fit obtained with the activity coefficients corrected after Davies [15]. The blue line is the population estimate from a mixed model with random intercept. The intercept is not significantly different from 0 and the slope not different from 1.

Employing the activity coefficient as described by Davies [15] resulted in a good fit with intercept 0.0156 ± 0.1094 i.e. 95% CI -0.199 to 0.230 and therefore not statistically different from zero, and slope of 0.9952 ± 0.0148 i.e. 95% CI 0.966 1.024 which is not significantly different from 1. This is shown in Fig 4.

Here the points in scatterplots and the lines of identity are unbiased whereas the lines representing the population mean from the mixed modeling are biased by the assumption that pH is measured without error. For the data represented in Fig 4 we also made a unbiased paired t-test. As expected with the large sample the difference was significant (P < 0.001), but the 95% confidence interval of the difference was quite narrow (-0.0136 to -0.0069) and close to zero.

### Sensitivity analysis

We performed a sensitivity analysis to confirm the ability of Eq 7 to inform on changing values of the variables. At the modeled pH, net charge as measured by the left hand side of Eq 1 was between -5*10^-17^ and 5*10^-17^ i.e. effectively zero as required. If [H^+^] was allowed to increase by 10% from the modeled value, net charge increased by 0.0027 (mean)—(0.0008–0.0048, range). This was similar to the finding if SID was allowed to increase by 10%: 0.0037 (mean)– 0.0014–0.0060 (range). Changes of the same order were observed with 10% change in pCO_2_ and albumin concentration.

### Nonparametric and Bland-Altman analysis

**Fig 5:**
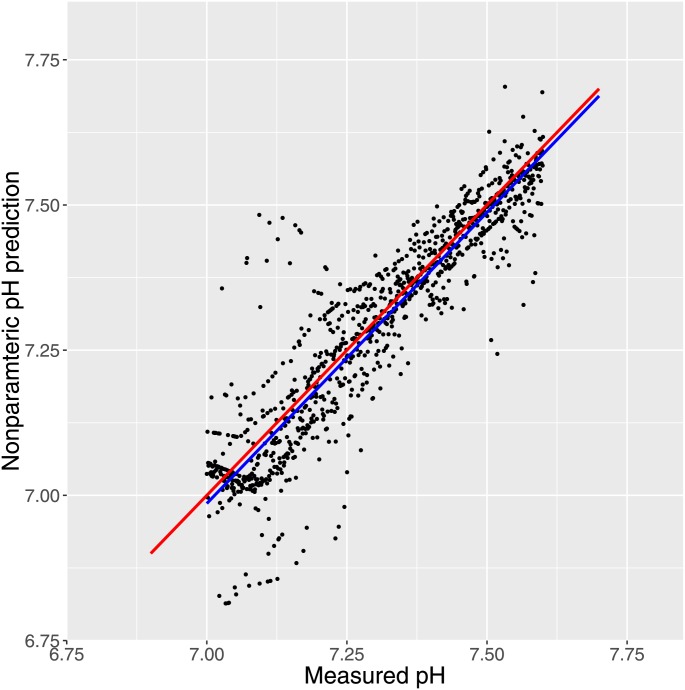
Fig 5 shows the prediction obtained by unbiased nonparametric regression (blue line) in comparison with the line of identity (red).

Also using the SIMEX algorithm [21] to correct for error in measured pH (ignoring the hierarchical nature of data) yielded intercept .930 ± 0.129 and slope 1.125 ± 0.017, both statistically significantly different from 0, P < 0.001 on data corresponding to Fig 4. This can be compared to the linear regression on the same data yielding intercept -0.574 and slope 1.077 and very similar standard errors. Furthermore we obtained unbiased prediction from a nonparametric kernel-based estimation [22]. Some results are presented in Fig 5.

**Fig 6:**
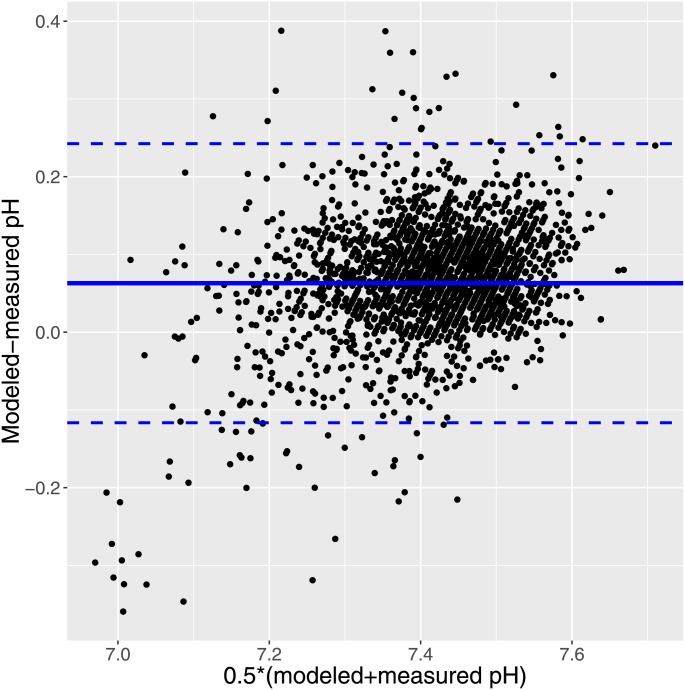
Fig 6 shows a Bland-Altman plot of fitted-measured pH versus mean of fitted and measured pH. The lines indicate mean and 95% confidence interval.

Also, a Bland-Altman plot of the concentration based fit is provided in Fig 6 indicating good agreement between measured and modeled pH, and no relationship to the average.

### Analysis of difference between modeled and measured pH

Fitting the concentration of an unknown weak acid with pKa 7 yielded a perfect fit for 2183 measurements, with median concentration of 7 mM, and the remaining 254 were perfectly fitted with a weak base with pKb 6 and median concentration of 5 mM. The concentration of fitted unmeasured weak acids was significantly negatively correlated with pH (P<0.001) but the effect was very small (correlation -0.14), and the same was true for unmeasured weak base. Also, we found for all samples the necessary delta SID to yield a perfect match between modeled and measured pH. Median delta SID was -4 mM, almost equal to the mean value, with a range from -16.7 mM to 15.3 mM. 89% of the delta values were negative as expected since the modeled pH was higher than the measured pH.

We further examined the difference between modeled and measured pH. The median within-patient standard deviation of this difference was 0.044 (range 0.008 to 0.238), and the median coefficient of variation (standard deviation/mean) was -1.6 and unrelated to the length of the series (P = 0.94). Likewise, the difference between modeled and measured pH was unrelated to the length of the series for individual patients.

**Fig 7:**
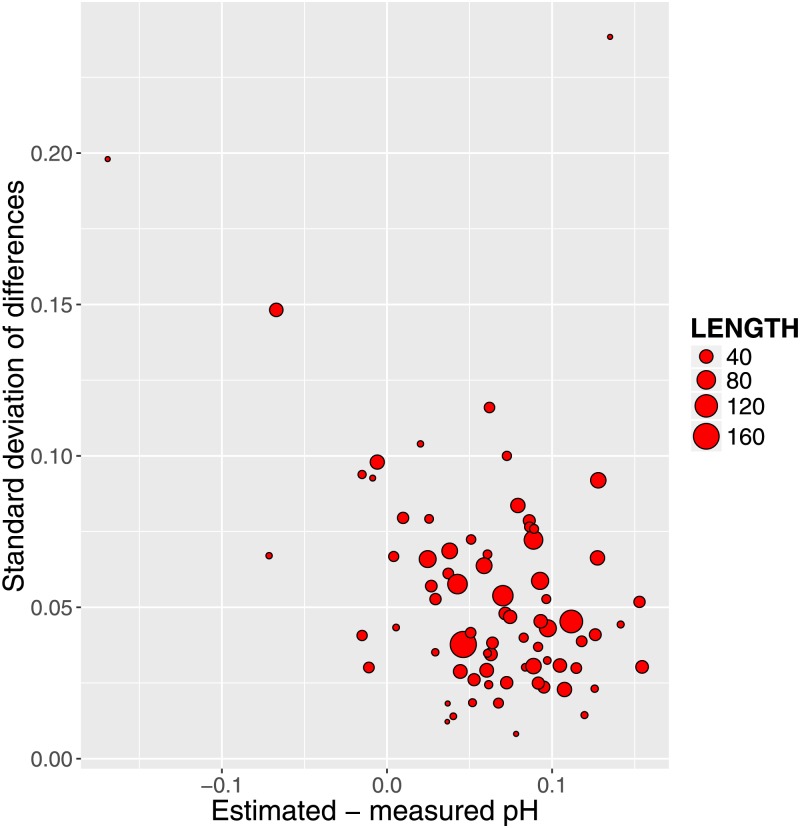
Fig 7 shows relationship between the standard deviations of the differences between modeled and estimated pH as a function of the corresponding means for the 76 patients. The area of the circles is proportional to sample size.

**Fig 8:**
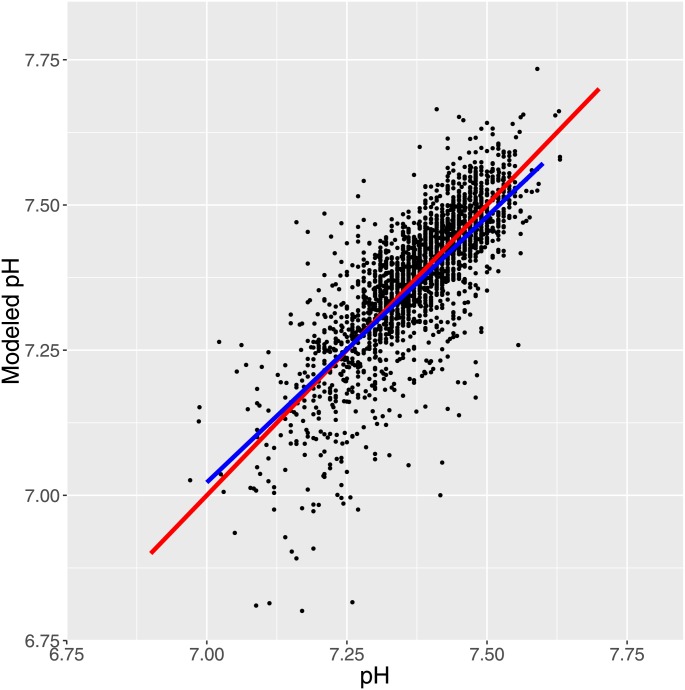
Fig 8 shows the fit obtained after assigning buffer with 6 mM pKa 7 to all samples represented by scatterplot, red line of identity and blue line of population mean.

We plotted (Fig 7) for the 76 patients the relationship between intra-patient standard deviation of differences between estimated and measured pH versus the corresponding mean values. The relatively homogeneous distribution could indicate that fitting a single correction for all patients might be feasible. To explore that possibility we employed a single fixed correction of 6 mM pKa 7 weak acid for all measurements and this appeared to be successful as shown in Fig 8.

**Fig 9:**
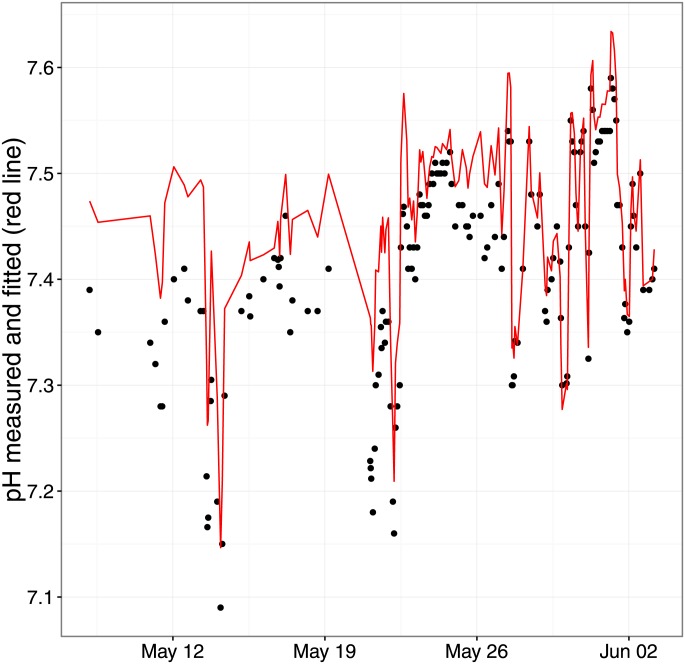
Fig 9 shows the time course of measured and modeled pH (red curve) in one of the longest series.

**Fig 10:**
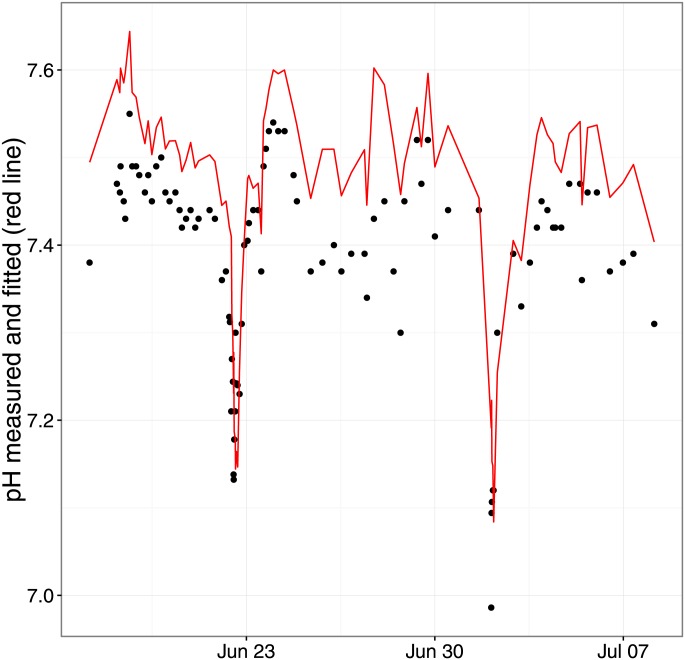
Fig 10 shows the time course of measured and modeled pH (red curve) in one of the longest series.

Plots of fitted and measured pH over time in two of the longest series are shown in Figs 9 and 10. It is evident, as indeed in all the series, that as measured pH fluctuates, modeled pH based on the 9 variables changes in close approximation—correlation in these series is 0.93 (95% CI: 0.90–0.95) between the two pH values.

### Independent validation of Eq 7

For the data from Pitts and Alexander [11] the mean value for observed titratable acidity was 0.242 mmol/min compared with 0.245 mmol/min as calculated based on Eq 7 (p = 0.893, 95% CI for difference -0.061 mmol/min to 0.054 mmol/min, and the correlation between the values was 0.999). Hence, from these old results further substantiation of the charge balance model of acid-base is gained.

## Discussion

The theoretical validity of Eq 7 is indisputable from physical chemistry, with the minor addition of charges on albumin as empirically verified by Watson [13]. However the demonstration that Eq 7 has utility in a clinical dataset is a demanding task. A very simple illustration of the problem on which we are focusing is presented in Figs 1 and 2, handling just the mixing 1:1 of two fluids, in which the [H^+^] cannot be predicted from averaging [H^+^] but is solely determined by SID.

We must emphasize that we are not attempting to devise a rather complicated way of determining pH by calculation based on a number of measurements since it would always be easier to measure pH directly, even if this has its own complexities [26]. In contrast, what motivated this analysis is the fact that if we can show that pH can be calculated with sufficient precision from the other variables which can be easily measured clinically, we can advance the notion that pH is mechanistically determined from these other charges. If Eq 7 is accepted as valid, a strong relationship between the charged moieties is imposed. This relation must be understood as causal because changes in the other charged moieties inevitably result in changes in [H^+^] and vice versa. This should pave the way for improved understanding of acid-base balance since SID constituents and total concentrations of weak acids behave predictably in contrast to [H^+^] [3] and bicarbonate [27].

To interpret the empirical results, we need first to consider how measurements of charges on say SID constituents with an error in the millimolar range can allow estimation of proton concentrations in a range several orders of magnitude lower. Noting in Eq 7 how the individual terms are dominated by proton concentrations and very small dissociation coefficients in the denominators explains why the equation of charge balance ends up being exquisitely sensitive to the protons concentrations thereby allowing their determination with reasonable precision. This is indeed confirmed by our sensitivity analysis since a 10% change in [H^+^] resulted in a similar effect on the left hand side of Eq 7 as a 10% change in SID.

Given the nature of our patients’ data including errors in all the measurements and the fact that concentrations of SID components and the measured weak acids were linearly interpolated to the time of the pH and pCO_2_ measurements, we should expect a large scatter and individual error in our results. As seen in the figures, including the Bland-Altman plot in Fig 6, this is indeed the case.

Also Eq 7, being based on the modeling of the albumin effect as described by Watson [13], assumes control of pH dependent interaction between albumin and calcium and magnesium. Although the free charge on albumin has been used to measure the concentration of albumin [28] we cannot assume this always to be straightforward. As an example, the binding of calcium to albumin has been found to decrease in uremia [29]. However, the net effect of this conundrum to perturb the modeling of pH is not likely to be great.

As a first step in presenting the results, unbiased scatterplots of modeled pH as a function of measured pH demonstrated strong empirical correspondence with the unmodified results and even more if activity coefficients [15] were imposed (Figs 3 and 4). For the latter we found a very narrow 95% interval of confidence (-0.0136 to -0.0069) strongly indicating that the measured pH was very well predicted by the modeled pH, although, as expected with such a large sample there was a statistically significant (but physiologically and clinically irrelevant) difference (P < 0.001).

Fitting the line representing the mean population from the mixed models employed was biased by possible measurement error in measured pH. It is certainly not the case that pH can be measured without error [26,30,31]. This obviously does not affect the scatter plots, but to take the problem into account we fitted nonparametric models. First employing the SIMEX [20] algorithm we demonstrated that the formal relationship from the linear models between estimated and measured pH was not an artefact induced by ignoring error in measured pH. For practical reasons, however, this assessment ignored the hierarchical nature of the data. Therefore we also assessed the relationship between measured and modeled pH by means of non-parametric kernel-based estimation [22], which took the clustering of data into account. By this method we found that predicted values were very close to measured values (Fig 5). In this situation, measurement errors in pH were unable to bias the results by sums of squares calculations as occurs in simple regression models. Also the Bland-Altman plot indicated a reasonable agreement between modeled and measured pH.

The ability of the correction of activity coefficients according to Davies [15]to improve the modeling as is evident in comparing Figs 3 and 4 is quite encouraging. Also the previously obtained very accurate prediction of measured pH was also obtained with correction for ionic strength [12]. Nevertheless, the theory of electrolyte solutions cannot be regarded as definite [32] so we will focus in the rest of the discussion primarily on the results obtained with uncorrected input measurements.

For the patients examined here it was evident that there was a statistically significant and physiologically important offset whereby fitted pH as built on concentrations overestimated measured pH. We established that this was not due to random error and we therefore sought to examine possible explanations for the deviation from measured pH. It has indeed been suggested that our knowledge in translating measured concentrations to true activities and in modeling ionic interactions at large is far from compelling [14], although the activities obtained a.m. Davies [15] apparently were able to remove most of the offset. It is notable that obtaining consistent measurements of pH that are lower than the modeled values has been reported previously and referred directly to the physical chemistry [26].

Setting the activity coefficient corrected data aside, we focused rather on the possibility that our patient plasma contained molecular species not accounted for. These patients were after all critically ill and in such patients unmeasured anions are frequently found in contrast to healthy persons [33] and perhaps more so when there is acidosis [34].

Our results demonstrate that by modifying Eq 7 by a single term specifying unmeasured weak acid or weak base we were able to obtain a perfect fit for all measurements with reasonable values of concentrations and dissociation coefficients for the postulated missing moieties. Also, as shown in Fig 8, assigning all samples a common weak acid with concentration 6 mM and pKa 7 apparently improved the correspondence between measured and modeled pH.

One appealing feature of the method used here is that it would allow direct tests of the importance of any mixture of specified weak acids or bases but obviously it was not possible to go further with this approach without access to blood samples from these patients. Likewise there could be problems in measuring SID components and to see if that alone could explain the offset we fitted a value of SID to give perfect match and saw that this value was median 4 mM too high.

Furthermore, we must also consider the possibility of sampling bias. The data used was from existing electronic medical records and it was impossible to confirm in more detail any hypothesis for the lack of exact fit. Also, for practical reasons, we only used a very small part of the vast material available. It is possible that patients with longer stay and more severe disease were overrepresented in the sample extracted since they were more likely to have enough values to be picked up. However we found no relationship between the length of the series and the deviation between modeled and measured pH. For this reason, and for the reasons given above, we find it unlikely that our interpretation that Eq 7 can yield useful information about pH is restricted to just these severely ill patients.

Finally, individual perfect modelling for every measurement can be achieved by very modest tweaking of the model by addition of a weak acid with plausible characteristics on top of the other measurements. This is not a statistical exercise but a direct mathematical operation. This is strong evidence of the robustness and versatility of the physiological model underlying Eq 7. In addition, we have previously shown that the pH of mixtures of solutions containing different electrolyte concentrations and buffers can be *exactly* predicted using equations similar to the one used here [12]. Similar results have been reported for humans under maximal exercise [16] and animals given fluid resuscitation [35].

Notwithstanding residual numerical reservations, it is very encouraging to see that for individual patients, fluctuations in measured and fitted pH coincided quite well as shown in Figs 9 and 10.

On the basis of these findings we conclude that we have provided considerable empirical support for Eq 7 as we attempted.

Independent support for the validity of Eq 7 is also derived from the analysis of the data from Pitts and Alexander [11]. As shown in the results section, Eq 7 allowed estimation of two SID values at urine and plasma pH, from which a very good estimate of measured titratable acidity was obtained. This indicates that the estimate of SID from Eq 7 is appropriate at the accuracy required. These data were the subject of editorial focus in American Journal of Physiology [36] because of their importance in developing the understanding of the way acid excretion occurs in the kidney. Therefore we find it very exciting that the quantitative results from Pitts and Alexander [11] can be exactly captured in our acid-base discourse as an example of coherence in modeling acid-base balance.

It is clear that Eq 7 allows determination of any single variable given the others, and thereby any dichotomy between independent and dependent variables is unnecessary. Hence, given that this equation, which was derived *a priori* from physical chemistry, has now been empirically validated, we can conclude that there is a strong relationship between all charged moieties in any fluid compartment.

The nature and implications of this relationship between charged species in a fluid have been debated for years. Stewart [4] made the proposal that the other charged species in Eq 7 *determined* [H^+^]. Siggaard-Andersen wrote in 1995: “Due to the electro-neutrality principle changes in the hydrogen ion status automatically involve changes in the electrolyte status but the opposite need not be the case” [37]. On the contrary, if Eq 7 is accepted, the relationship between pH and SID is strong and mechanistic and modulated by the other factors in an explicit way. It is indeed true that SID reflects the difference between added strong base and strong acid [38]—this difference is not directly evident in pH in biological solutions. This was clear to Spiro and Koppel in 1914 in their statement that protons were not additive [3] as demonstrated in our simulations (Figs 1 and 2). Therefore, the effect of SID on pH is the combined effect of strong base and acid, which few would suggest should be ignored even if it may be hard to accept that Eq 7 (or Eq 2) is needed to quantify it.

The insight that there is a strong and predictable relationship between the concentrations of charged moieties is not new [3] at all and certainly not outlandish [1].

In classic experiments, SID components (Cl^-^) were observed to be necessary to balance fluxes of protons in the collecting duct [39], and in normal people, sodium chloride intake had an independent and classically unexplained effect on acidosis [40]. The authors [40] mentioned that a calculation of SID resulting from increasing sodium chloride intake could explain the findings. In a 1955 paper by Giebisch and Pitts [41], extrarenal buffering to respiratory acid-base disturbances was accounted for by SID components and weak acids:

1. Transfer of chloride across the erythrocyte membrane;
2. shift of sodium and potassium across an extracellular boundary other than the red cell membrane;
3. transfer of inorganic phosphate across an unknown extracellular boundary; and
4. *significant alterations in lactate metabolism*.

All these examples demonstrates that there has been in classical physiology a strong respect for the requirements imposed by physical chemistry and charge balance on the movements of ions and protons. Our intention is to revive this tradition. The demonstration using the data from Pitts and Alexander [11] that the estimate of SID as obtained from Eq 7 is valid is a first demonstration that we should think of strong ions when trying to understand the renal involvement in acid-base regulation [2].

One limitation of our study is that we were unable to provide a detailed account of unmeasured species in order to prove that we could model pH increasingly well. However, we should never expect to be able to prove a theoretical equation exactly right by empirical analyzes of clinical samples however complete they are. Nevertheless, the perfect fit obtained under laboratory conditions [12] and the very good fit obtained with correction activity coefficients [15], provide considerable support for the construct in addition to its derivation from physical chemistry. One next step is to show that the modeling of acid-base physiology based on strong ions overcomes the limitations of the conventional model in particular in accounting for the renal effect on acid-base balance [42,43] or the effects due to fluid resuscitation or extracorporeal therapies (e.g. dialysis). Theoretical and experimental work in this respect is in progress.

In conclusion, our study provides experimental validation of Eq 7, which establishes a strong and mechanistic relationship between strong ions and acid-base. Eq 7 should therefore be a useful tool to explore acid-base relationships in physiology and medicine. Looking back to classical physiology, we have given a number of illustrations of the utility of this construct.

## Supporting Information

S1 Text Derivation of equations. (DOCX)
