# Supplementary material for: Strong Relationships in Acid-Base Chemistry – Modeling Protons Based on Predictable Concentrations of Strong Ions, Total Weak Acid Concentrations, and pCO2
Source: PLoS One. 2016 Sep 15;11(9):e0162872. doi: 10.1371/journal.pone.0162872 (PMC5025046; doi:10.1371/journal.pone.0162872)
Supplement: S1 Text — (DOCX) [file pone.0162872.s001.docx]

Derivation of Equations

## Water dissociation

$$k_{w}=\left[H^{+}\right]*\left[OH^{-}\right]$$

## Monovalent weak acid

$$\begin{matrix} \left[H^{+}\right]*\left[A^{-}\right]=k_{a}*\left[HA\right] \\ \left[Atot\right]=\left[HA\right]+\left[A^{-}\right] \\ \left[A^{-}\right]=\frac{k_{a}*\left[Atot\right]}{k_{a}+\left[H^{+}\right]} \end{matrix}$$

The charge on a number of weak acids then is the sum of their individual charges

## Derivation of charge on P as a function of total concentration and pH

$$\left[HPO_{4}^{--}\right]+\left[H^{+}\right]\rightleftarrows\left[H_{2}PO_{4}^{-}\right]$$

$$k_{2}=\frac{\left[H^{+}\right]*\left[HPO_{4}^{--}\right]}{\left[H_{2}PO_{4}^{-}\right]}$$

$$P=\left[HPO_{4}^{--}\right]+\left[H_{2}PO_{4}^{-}\right]$$

This is an approximation but very little error is committed within reasonable pH values between 4 and 10 by ignoring trivalent phosphate and undissociated phosphoric acid[[1]](#endnote-1)

$$\left[H_{2}PO_{4}^{-}\right]=P-\left[HPO_{4}^{--}\right] \Leftrightarrow k_{2}=\frac{\left[H^{+}\right]*\left[HPO_{4}^{- -}\right]}{P-\left[HPO_{4}^{- -}\right]}$$

$$k_{2}*P-k_{2}*\left[HPO_{4}^{- -}\right]=\left[H^{+}\right]*\left[HPO_{4}^{- -}\right] \Leftrightarrow k_{2}*P=\left(\left[H^{+}\right]+k_{2}\right)*\left[HPO_{4}^{- -}\right]$$

$$\left[HPO_{4}^{--}\right]=\frac{k_{2}*P}{\left[H^{+}\right]+k_{2}}$$

Charge on divalent species therefore is $\frac{2*k_{2}*P}{\left[H^{+}\right]+K_{2}}$

Univalent P:

$$\left[H_{2}PO_{4}^{-}\right]=P-\left[HPO_{4}^{--}\right]=P-\frac{k_{2}*P}{\left[H^{+}\right]+k_{2}}=\frac{P*H}{\left[H^{+}\right]+k_{2}}$$

So total charge is $\frac{2*k_{2}*P}{\left[H^{+}\right]+k_{2}}+\frac{P*H}{\left[H^{+}\right]+k_{2}}=\frac{P*(k_{2}+k_{2}+\left[H^{+}\right])}{\left[H^{+}\right]+k_{2}}=P*\left[1+\frac{k_{2}}{\left[H^{+}\right]+k_{2}}\right]$

## Weak base

$$\left[B\right]+ H_{2}O \rightleftarrows\left[BH^{+}\right]+\left[OH^{-}\right]$$

$$\left[Btot\right] = \left[B\right]+ \left[BH^{+}\right]$$

$$k_{b} = \frac{\left[BH^{+}\right]*\left[OH^{-}\right]}{\left[B\right]} = \frac{\left[BH^{+}\right]*\left[OH^{-}\right]}{\text{ }\left[\text{Btot}\right]\text{-}\left[\text{BH}^{+}\right]\text{ }}$$

$$\begin{matrix} \left[BH^{+}\right]*\frac{k_{w}}{\left[H^{+}\right]} = k_{b}*\left[Btot\right]-k_{b}*\left[BH^{+}\right] \\ \\ \left[BH^{+}\right] = \frac{k_{b}*\left[Btot\right]}{k_{b}+\frac{k_{w}}{\left[H^{+}\right]}}= \frac{k_{b}*\left[Btot\right]*\left[H^{+}\right]}{\left[H^{+}\right]*k_{b}+k_{w}} \end{matrix}$$

So the charge of a number of weak bases is the sum of their individual charges

## Charge on carbonates

$$\begin{matrix} \left[H_{2}CO_{3}\right] \rightleftarrows\left[H^{+}\right]+\left[HCO_{3}^{-} \right] \Leftrightarrow k = \frac{\left[H^{+}\right]*\left[HCO_{3}^{-} \right] }{\left[H_{2}CO_{3}\right]} \\ k_{c} = \frac{\left[H^{+}\right]*\left[HCO_{3}^{-} \right] }{pCO_{2}}\Leftrightarrow\left[HCO_{3}^{-} \right] = \frac{k_{c} *pCO_{2} }{\left[H^{+}\right]} \\ \left[H^{+}\right]*\left[CO_{3}^{--} \right] =k_{3}*\left[HCO_{3}^{-} \right] \Leftrightarrow\left[CO_{3}^{--} \right] =\frac{k_{3}*\left[HCO_{3}^{-} \right]}{\left[H^{+}\right]} = \frac{k_{3}*k_{c}*pCO_{2} }{\left[H^{+}\right]^{2}} \end{matrix}$$

Hence the charge on CO_3_^- -^ is $2*\frac{k_{3}*k_{c}*pCO_{2} }{\left[H^{+}\right]^{2}}$

## Charge on albumin

Watson found an effective modeling of the charge on albumin by taking 21 fixed negative charges per molecule, so the charge here is $Alb*AF$with Alb the molar concentration and AF = 21

Further, since each molecule contains 16 histidine residues, these were modeled by

$[H^{+}]*Alb*AH/(k_{h}+[H^{+}])$with AH = 16 and k_h_ 1.77e-7

So putting it all together, and put summation on weak acids and bases, we have equation 1

$$\begin{matrix} \left[H^{+}\right]+SID-\frac{k_{w}}{\left[H^{+}\right]}-\frac{k_{c}*pCO_{2}}{\left[H^{+}\right]}-\frac{2*k_{3}*k_{c}*pCO_{2}}{\left[H^{+}\right]^{2}}-P*\left(1+\frac{k_{2}}{k_{2}+\left[H^{+}\right]}\right)- \\ Alb*AF+\left[H^{+}\right]*Alb*\left(\frac{AH}{k_{h}+\left[H^{+}\right]}\right)-\sum\frac{k_{a}*Atot}{k_{a}+\left[H^{+}\right]}+\sum\frac{\left[H^{+}\right]*k_{b}*Btot}{\left[H^{+}\right]*k_{b}+k_{w}}=0 \end{matrix}$$

1. Watson PD. Modeling the effects of proteins on pH in plasma. J Appl Physiol 1999; 61: 1444.1461

   ## Accommodating multivalence moieties

   As an example, phosphate can be taken as polyvalent (e.g. Kildeberg, Scand J clin lab invest 43;103-109, 1987) with 3 pK values and then instead of the P term use
   $P*\frac{10^{-\log_{10}(H)-pK_{1}}+2*10^{-2*\log_{10}(H)-pK_{1}-pK_{2}}+3*10^{-3*\log_{10}(H)-pK_{1}-pK_{2}-pK_{3}}}{1+10^{-\log_{10}(H)-pK_{1}}+10^{-2*\log_{10}(H)-pK_{1}-pK_{2}}+10^{-3*\log_{10}(H)-pK_{1}-pK_{2}-pK_{3}}}$

   [↑](#endnote-ref-1)
